# Editorial on the Advances in Organic Synthesis in Pharmaceuticals, Agrochemicals and Materials

**DOI:** 10.3390/molecules30214163

**Published:** 2025-10-23

**Authors:** Jiuzhong Huang, Zhao-Yang Wang

**Affiliations:** 1 Jiangxi Province Key Laboratory of Pharmacology of Traditional Chinese Medicine, School of Pharmacy, Gannan Medical University, Ganzhou 341000, China; huangjz@gmu.edu.cn; 2 School of Chemistry, South China Normal University, Guangzhou 510006, China

This Special Issue, entitled “Advances in Organic Synthesis in Pharmaceuticals, Agrochemicals and Materials”, offers a comprehensive overview of the latest progress and multifaceted applications in organic synthesis. It showcases the intricate science and artistry involved in molecular construction and highlights the field’s profound impact across multiple disciplines.

A central theme of this collection is the development of novel synthetic methodologies that enable precise and controlled assembly of complex molecular architectures. These advances not only improve reactivity and selectivity but also open new pathways for producing pharmaceuticals, agrochemicals, and functional materials [1,2,3,4,5,6]. The eight selected contributions exemplify this evolution, presenting innovative approaches—including synthetic methodology, element organic chemistry, natural product total synthesis, and biological activity of small molecules—that enrich the synthetic toolkit while offering mechanistic insights and potential applications in catalysis, medicinal chemistry, and materials science [7,8,9,10].

Firstly, Ye et al. (2024) report a molybdenum-catalyzed anti-Markovnikov hydrosilylation of alkynes with high (E)-selectivity [11]. Using a commercially available Mo(CO)_6_/dppb catalyst system under mild conditions, the method exhibits excellent functional group tolerance and regioselectivity. This work offers a cost-effective and sustainable alternative to noble-metal catalysts, with a proposed mechanism involving oxidative addition, migratory insertion, and reductive elimination supported by control experiments. The next contribution, by Ashirov et al. (2025), describes the synthesis and characterization of hydrogen-bonded di(hydroperoxy)alkane adducts with tricyclohexylphosphine oxide (Cy_3_PO) [12]. These shelf-stable, crystalline compounds display high solubility in organic solvents and act as efficient oxidants, as shown in the rapid oxidation of triphenylphosphine. Structural and spectroscopic analyses have confirmed that hydrogen bonding weakens the P=O bond. This weakening enhances the compound’s oxidative reactivity. Consequently, the solid peroxides now have new and promising possibilities for use in synthesis. In the third paper, Xu et al. (2025) investigate the dual role of N, N-dimethylformamide (DMF) in copper-catalyzed domino reactions toward Se-phenyl dimethylcarbamoselenoates [13]. Starting from aryl halides and selenium powder, the reaction proceeds under mild conditions with a broad substrate scope. Deuterium labeling and mass spectrometry studies indicate oxidative addition as the rate-determining step, offering an economical route to valuable organoselenium compounds. Liu et al. (2025), in the fourth paper, present a one-pot Heck/dehydration strategy for the total synthesis of trans-dehydroosthol and citrubuntin—key meroterpenoid intermediates [14]. By employing less reactive bromocoumarins and optimized base/solvent systems, the authors achieved high yields and stereoselectivity without protecting groups. This redox-neutral and atom-economical approach streamlines access to complex natural product frameworks.

The fifth contribution revisits the bioactivity of shikonin, previously identified as a strong SARS-CoV-2 Mpro inhibitor [15]. Cross-validation studies, however, revealed that inhibition occurred only in the absence of reducing agents such as DTT, suggesting shikonin acts as a non-specific cysteine protease inhibitor with associated cytotoxicity. Reactive oxygen species generation and bioreductive alkylation were implicated in its off-target toxicity. These findings highlight the need to modify the naphthazarin scaffold to develop specific Mpro inhibitors with improved safety profiles. In the sixth study, twenty novel 5-(1-amino-4-phenoxybutylidene)barbituric acid derivatives bearing an enamino diketone motif were synthesized and evaluated for herbicidal activity [16]. One compound, BA-1, demonstrated promising efficacy, a broad herbicidal spectrum, and good crop safety. Structure-activity relationship (SAR) studies indicated that steric, electronic, and lipophilic properties significantly influence activity. Molecular docking suggested that BA-1 binds effectively to NtPPO, indicating potential as a novel PPO inhibitor and a candidate for further optimization.

The seventh paper highlights disubstituted Meldrum’s acid as a new carbon-based scaffold with sulfur(VI) fluoride exchange (SuFEx)-like reactivity [17]. Phenols proved optimal nucleophiles, while thiols and thiophenols—often problematic in classical SuFEx—exhibited comparable reactivity. Alcohols and amines required elevated temperatures, and sterically hindered nucleophiles remain challenging, motivating ongoing catalyst development. Lastly, Guest Editor Prof. Wang and Dr. Huang developed a nickel-catalyzed [2 + 2 + 2] cycloaddition of alkynes, nitriles, and allyl boronates [18]. This concise catalytic system allows efficient, regioselective synthesis of fused pyridine derivatives from readily available materials. Mechanistic studies underscored the essential role of the terminal double bond and the Bpin group.

As of 13 October 2025, these eight articles have collectively attracted 8303 views, averaging 1038 views per publication. Together, these studies underscore the critical roles of catalyst design, solvent participation, and reaction engineering in modern synthetic chemistry. They also reflect a shift toward sustainability—employing earth-abundant metals, stable oxidants, and domino processes to reduce waste and synthetic steps [19,20,21]. By bridging organic, inorganic, and materials chemistry, this Issue embodies the collaborative and interdisciplinary spirit driving innovation in the field.

We commend the authors for their contributions and encourage continued exploration into the mechanisms and applications of these promising methodologies.
